# Photometric Sensing of Active Chlorine, Total Chlorine, and pH on a Microfluidic Chip for Online Swimming Pool Monitoring

**DOI:** 10.3390/s20113099

**Published:** 2020-05-30

**Authors:** Sait Elmas, Aneta Pospisilova, Aneta Anna Sekulska, Vasil Vasilev, Thomas Nann, Stephen Thornton, Craig Priest

**Affiliations:** 1 Future Industries Institute, University of South Australia, Mawson Lakes, SA 5095, Australia; sait.elmas@flinders.edu.au (S.E.); pospisilova-a@seznam.cz (A.P.); aneta.sekulska@gmail.com (A.A.S.); vasil.rosenov.vasilev@gmail.com (V.V.); Thomas.Nann@newcastle.edu.au (T.N.); 2 Institute for Nanoscale Science & Technology, College of Science & Engineering, Flinders University, Sturt Road, Bedford Park, SA 5042, Australia; 3 School of Mathematical and Physical Sciences, University of Newcastle, University Drive, Callaghan, NSW 2308, Australia; 4 Tekelek Australia Pty Ltd., 95A Bedford St, Gillman, SA 5013, Australia; sthornton@tekelek.com.au; 5 School of Engineering, University of South Australia, Mawson Lakes, SA 5095, Australia

**Keywords:** chlorine, pH, microfluidic, photometric, online, swimming pool

## Abstract

A microfluidic sensor was studied for the photometric detection of active chlorine, total chlorine, and pH in swimming pool samples. The sensor consisted of a four-layer borosilicate glass chip, containing a microchannel network and a 2.2 mm path length, 1.7 μL optical cell. The chip was optimised to measure the bleaching of methyl orange and spectral changes in phenol red for quantitative chlorine (active and total) and pH measurements that were suited to swimming pool monitoring. Reagent consumption (60 μL per measurement) was minimised to allow for maintenance-free operation over a nominal summer season (3 months) with minimal waste. The chip was tested using samples from 12 domestic, public, and commercial swimming pools (indoor and outdoor), with results that compare favourably with commercial products (test strips and the N,N′-diethyl-p-phenylenediamine (DPD) method), precision pH electrodes, and iodometric titration.

## 1. Introduction

Chlorination is widely used to disinfect recreation and drinking water, is strictly regulated [1,2], and must be carefully monitored to avoid outbreaks of waterborne disease [3,4]. While commercial water treatment is professionally managed by water utilities and the government, most swimming pools are managed by non-technical pool owners (domestic) or operators (commercial/public), who rely on retail test products and “grab” (manual) sampling. Most online alternatives are expensive, require regular maintenance, and are difficult to retrofit into an existing pool. Here, we address the demand for a chip-based photometric chlorine (active and total) and pH sensor that is suitable for online monitoring on domestic and commercial/public swimming pools.

Active chlorine can be produced upon the aqueous dissolution of inorganic hypochlorite salts, photocatalytically on Ag@AgCl and TiO_2_ photoelectrodes [5,6,7,8,9], or directly via generation of molecular chlorine (Cl_2_) at an electrode–water interface [10,11,12,13].

Chlorine gas that is generated at an anode surface immediately disproportionates in swimming pool water to form HOCl and HCl, acidifying the water. Active chlorine is photolytically cleaved upon exposure to ultraviolet radiation (i.e., sunlight), and is consumed in redox reactions with other components of pool water, such as organic matter or nitrogen compounds. Active chlorine can also react with nitrogen-containing organics (urea, sweat, and microorganisms) to form chloramines, termed “bound” chlorine. Chloramine is also a disinfection agent but remains in the solution for longer than HOCl and has an inferior disinfection power. The sum of active and bound chlorine, “total” chlorine, is typically reported alongside active chlorine as one of the two critical indicators of swimming pool health.

The disinfection power [14] of active and bound chlorine species and their stability [15,16,17] in water strongly depend on the pH. Yet, the above reactions vary in pH over time (due to the acidity of HOCl) and swimming pools must be buffered, usually with sodium bicarbonate. Therefore, active chlorine, total chlorine, and pH must be monitored regularly [15,16,17]. Highly frequented swimming pools are ever-changing environments, with bacteria, other microorganisms, sweat, saliva, and urine being introduced daily, making water quality transient and management difficult. The under-dosage of chlorine creates risks of infection among swimmers, while over-dosage may cause adverse effects to the skin, eyes, and immune system [18,19,20].

Effective swimming pool management and safety demands a continuous and automated water quality sensor that can measure (at least) active chlorine, total chlorine, and pH. Most existing technologies are expensive, require maintenance, and many domestic pool owners still rely on test strips and other manual low-cost sensors. Progress in water monitoring is being made by groups using photometric sensors for the reliable chemical analysis of environmental water [21,22]. Functional materials (e.g., quantum dots) have also been used in the photometric method to enhance sensitivity to chlorine [23,24]. Nonetheless, the reliable analysis of real-world samples remains a challenge and, for this reason, the present study included samples from domestic, commercial, and public pools.

**Figure 1:**
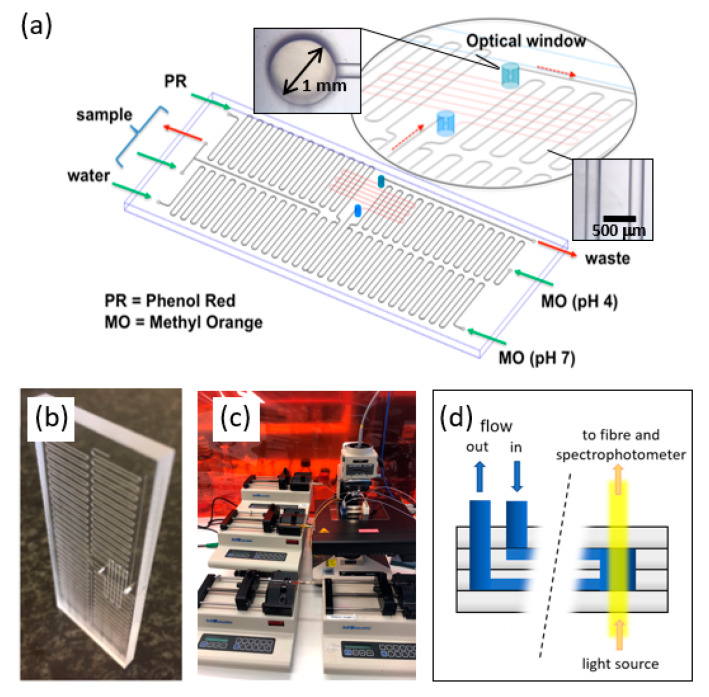
(**a**) Microfluidic chip for swimming pool sensing of active chlorine, total chlorine, and pH. Inset: the through-hole and optical window that creates the fluid connections between layers. (**b**) Photograph of the thermally bonded, four-layer borosilicate chip. (**c**) Setup used in this study. (**d**) Illustration of flow and light paths through the four glass layers of the chip.

Here, a lab-on-a-chip sensor, capable of addressing the above challenges, is investigated, as shown in Figure 1. The chip uses the photometric analysis of methyl orange and phenol red solutions in a 2.2 mm path length optical cell in a borosilicate glass chip. The estimated reagent use over a season (e.g., 3 months) is less than 35 mL, depending on operational protocols. Samples from 12 swimming pools were analysed, giving results that compare well with commercial products (test strips and N,N′-diethyl-p-phenylenediamine (DPD) tablets), pH electrodes, and iodometric titration. In domestic pools, the severe over-dosing and under-dosing of active chlorine was discovered, including one example where inaccurate testing at a retail outlet had misinformed the owner. This highlights the need for an accurate and reliable online pool sensor.

## 2. Materials and Methods

### 2.1. Materials

Sodium hypochlorite containing 4.00–4.99% available chlorine, phenol red (ACS grade) and starch (ACS grade) were purchased from Sigma Aldrich and used without further purification. Methyl orange, anhydrous citric acid, potassium iodide, sodium hydrogen bicarbonate, and sodium thiosulfate were obtained from Chem Supply as analytical grade materials. Glacial acetic acid (Chem Supply, 80%), sodium bromide (Scharlau, extra pure), and all other chemicals were used as obtained from the supplier. Deionised (DI) water (Milli-Q^®^ Advantage A10 Water Purification System, Merck Millipore) was used to dilute all samples and dye solutions to the required concentrations.

For the active chlorine analysis, 100 ppm MO stock solution containing 1000 ppm NaBr was used. The solution is further referred to as MO (pH 7). For the total chlorine analysis, buffered, acidified 100 ppm MO stock solution containing 4000 ppm NaBr was used. The solution was prepared from 90 mL of 63 mM citrate buffer (pH = 4) and 10 mL of 1000 ppm aqueous solution of MO. In the following discussion, the solution is referred as MO (pH 4).

Calibration lines for active and total chlorine were obtained by measuring sodium hypochlorite solutions containing 0–10 ppm of hypochlorite. Procedures for the measurements were the same as described below for real samples. The calibration solutions were prepared by dissolving aliquots of 470 ppm NaOCl in 50 ppm NaHCO_3_. The 470 ppm NaOCl solution was prepared by the dilution of commercially available sodium hypochlorite containing 4.00%–4.99% available chlorine and was standardized using the iodometric method [25].

The phenol red (PR) solution was prepared by modifying a commercially available pH indicator dye solution (HYCLOR^®^ pH Phenol Red, Hyclor Australia Pty. Ltd., Australia) containing 4.5% chlorine quencher. The concentration of PR was optimised for use in the chip by diluting 2 mL pH indicator solution with 398 mL DI water and adding 16 mg solid PR (Sigma Aldrich). To help dissolve the solid PR, one drop of 1 M NaOH was added. A calculation of the concentration of PR in the prepared solution (pH 6.4) was 41.2 mg/L phenol red, based on the Beer–Lambert analysis.

Calibration points for the pH method were prepared by the adjustment of a randomly chosen swimming pool sample. Aliquots (20 mL) of the sample were adjusted to the required pH (monitored using a pH meter) by the addition of several drops of 0.5 M NaOH or 0.5 M HCl and were measured as described below for real samples.

Swimming pool samples were collected from 12 outdoor pools (nine domestic, two indoor public, and one outdoor public) located in metropolitan Adelaide, Australia. The three public pools are used by many people on a daily basis. The sample IDs are anonymous (numbered), as the identities and locations of the pools and donors are unimportant in this study. Samples (500 mL) were collected and stored at 4 °C until use.

### 2.2. Chip Design and Preparation

The use of photometric methods demands clear windows and a suitable path length for detection. In this study, a four-layer thermally bonded glass chip was used. The upper and lower layers provided the clear glass windows for the photometric sensing and the two middle layers contained through-holes (with one used as the optical cell, as shown in the Figure 1a inset), and the microchannel network. The design of the bonded chip is shown in Figure 1a, with an inset showing the details of the optical window and the surrounding channels.

The chip was prepared in borosilicate glass (Borofloat 33) via Cr/Au-photoresist masking, followed by wet etching using 50% hydrofluoric acid. The through-holes (optical cell and inlet/outlet ports) were laser machined. The masking materials were removed by cerium ammonium nitrate solution, iodine/iodide solution, and acetone for Cr, Au, and photoresist, respectively. Thermal bonding was achieved at 630 °C and 1.5 kPa. A custom chip holder was prepared in polymethylmethacrylate (PMMA) to allow for interfacing with the FEP tubing.

The high flow resistance of the serpentine channels prevent backflow of the reagent and sample. These channels, and the outlet channel beyond the optical cell, have a cross-section of 103 μm × 214 μm. The channel in the lower layer of the chip (channel marked in red) immediately before the optical cell, allows diffusive mixing before detection of MO or PR in the optical cell, Figure 1a. This channel has a cross-section of 117 μm × 245 μm, and is 9.4 cm long. The optical cell has a path length of 2.2 mm and a diameter of 1 mm, i.e., 1.7 μL volume.

### 2.3. Off-Chip Experiments

**Figure 2:**
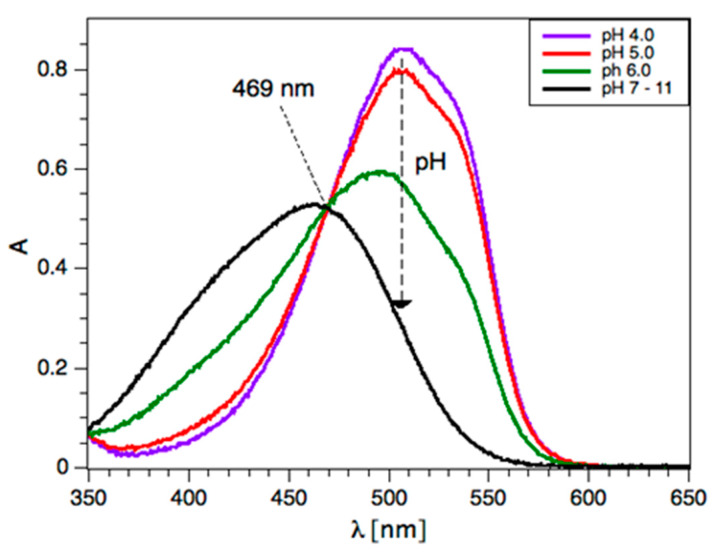
Isosbestic point for methyl orange. The spectra for pH 7–11 overlap and are represented by a single black line.

All experiments in the bulk (off-chip) were conducted at room temperature (R.T.). The collected swimming pool samples were stored at 4 °C and allowed to reach R.T. prior to their analysis. All of the dye solutions were kept at R.T. in the dark. Off-chip UV-Vis experiments were conducted in a 2 mm quartz cuvette (Ocean Optics, QE65000). For chlorine analyses, the detection wavelength was set at the isosbestic point of MO (469 nm) [26] to eliminate the bathochromic shift in the acidic pH regime, as shown in Figure 2. The methyl orange solution was pipetted into the cuvette before the sample was added and immediately mixed vigorously. For the 1:1 mixing ratio, 200 µL of sample and 200 µL of MO were used. For the 2:1 mixing ratio, 400 µL of sample and 200 µL of MO were used. For the 3:1 mixing ratio, 150 µL of sample and 50 µL of MO were used. For the 4:1 mixing ratio, 200 µL of sample and 50 µL of MO were used.

**Figure 3:**
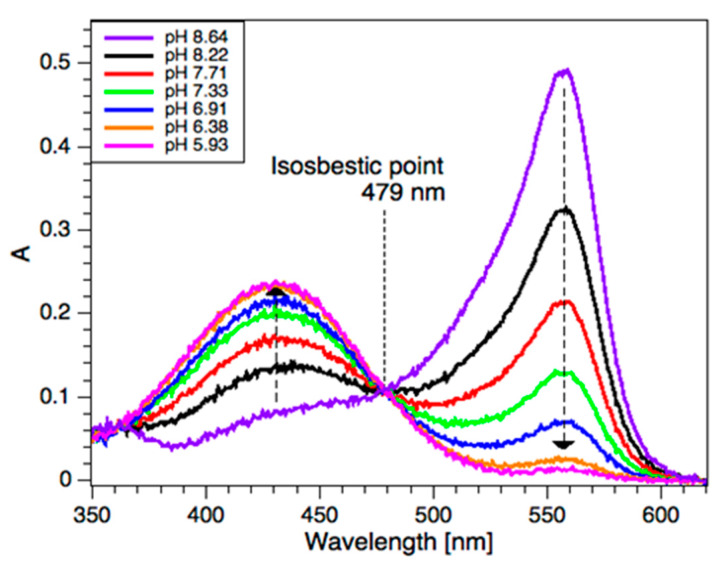
Phenol red absorbance spectra after mixing 1:1 with a pH-adjusted swimming pool sample.

The pH analysis in the bulk was conducted in a 2 mm quartz cuvette on the Ocean Optics spectrometer by mixing the sample and the PR stock solution in a 1:1 ratio (200 µL of sample and 200 µL of PR). The absorbance spectra for phenol red are given in Figure 3. The natural logarithm of the relative absorbance, A1/A2, 432/560 nm, was recorded. The pH was measured with pH meter OAKTON pH 2700 using an ORION 8220BNWP pH micro electrode (Thermo Fisher Scientific). The probe was calibrated against pH 4, 7, and 10 buffer solutions.

The total chlorine concentration was determined using iodometric titration, according to the international standard procedure [25] and was modified for low concentrations of chlorine in pool water. Our procedure follows the method for available chlorine, the only difference being the use of 0.5 mM sodium thiosulfate instead of 0.1 M. The solution was prepared by diluting 2.5 mL of the 0.1 M Na_2_S_2_O_3_ standard to 500 mL with deionised, freshly boiled, then cooled water. When standardizing against potassium iodate, our sodium thiosulfate solution was found to be 0.47 mM. Potassium iodide (2–3 g) and acetic acid (10 mL) were dissolved in 50 mL of water in a 250 mL Erlenmeyer flask and 50 mL aliquot of the sample was added. The sample was then titrated with the Na_2_S_2_O_3_ solution until the iodine colour was nearly gone. After adding 1 mL of the starch solution [25], the end point of the further titration with Na_2_S_2_O_3_ was found, when the blue colour of the starch disappeared. The titration was repeated three times and the average end point was used for the calculation of the total chlorine levels.

Universal test strips (AquaCheck^®^ AC7, HACH^®^, Loveland, CO, USA) were used by submerging the strips into the sample solution and matching the colour with the colour chart after 15 s reaction time.

The DPD powder (5 mL powder pillow, HACH^®^ Permachem Reagents) was added to 5 mL sample solution and shaken for 20 s. Then, the absorbance of the DPD solution at 530 nm was immediately recorded on the UV-Vis spectrophotometer (Ocean Optics) in a 10 mm quartz cuvette. The concentrations of the active chlorine levels were calculated from the calibration line. Calibration was found to be linear between 0–5 ppm. To analyse more concentrated samples, 1 mL of sample was diluted with 4 mL of DI water and then treated as described above. The result was multiplied by the dilution factor to obtain the original concentration of the sample.

### 2.4. On-Chip Experiments

On-chip experiments were conducted using the same reagent solutions and conditions as were used for the off-chip experiments. Samples and reagents were pumped using precision syringe pumps (KD Scientific) and 1 mL micro-syringes (Gastight^®^ Instruments Syringes, Hamilton, OH, USA). The streams merged upstream of the optical cell, mixed by diffusion, and were then measured through the optical window. Proof-of-principle experiments were successfully conducted using LEDs and photodiodes; however, the results presented here were obtained using a custom-built micro-spectrophotometer, based on an Olympus BH2-UMA frame [27]. This allowed the full spectrum (200–1000 nm) to be collected and inspected for potential interferences or anomalies (e.g., in real pool samples). In addition, the micro-spectrophotometer has an in-built digital camera that allows the user to visualise the channels and check for errors due to fouling or air bubbles.

On-chip measurements were collected using the protocol in Table 1. The protocol can be automatically repeated continuously or semi-continuously, depending on the maintenance requirements. A feedback loop would allow for the regular chemical dosing of the pool without human intervention (unlike with the use of test strips). The optimisation of dose amounts and frequency would reduce the cost and labour required to maintain the pool chemistry. First, the sample channel was flushed with 100 µL of sample. Then, for active chlorine analysis, MO (pH 7) was pumped together with the sample at the required flow rate ratio. The overall flow rate was 10 µL/min in all cases. For analysis of real samples, the mixing ratio 1:3 was used for samples containing less than 8 ppm active chlorine, and the 1:1 mixing ratio was used when the concentration was higher. Changes in absorbance at 469 nm and 650 nm (background) were observed using the OceanView strip chart. After the stabilizing of signals (<2 min), the absorbance was recorded for 4 min. The absorbance at 650 nm (background) was subtracted from that at 469 nm. The average values were used for calculating the concentration to minimize the influence of any transient instabilities.

After active chlorine analysis, the MO (pH 7) pump was turned off and the MO (pH 4) pump was turned on for total chlorine analysis. The protocol was the same as for the active chlorine analysis, described above.

After total chlorine analysis, the MO (pH 4) pump was turned off and the PR pump was turned on. The mixing ratio was 1:1 (5 µL/min sample and 5 µL/min PR). Absorbances at 432, 560, and 650 nm were observed in the OceanView strip chart. After the stabilizing of signals, absorbances were recorded for 4 min. The logarithm of relative peak intensity was calculated, ln((A432–A650)/(A560–A650)), and the average value was used to calculate the pH.

## 3. Results

### 3.1. Chip Function

First, the physical parameters of the device and their impact on the chip operation were considered. The three critical parameters were pressure drop, residence time, and diffusion time. The flow in the chip had to meet two key conditions: (1) the flow resistance in the sample and reagent inlet streams should be large enough to minimise backflow during operation; (2) the residence time from the merging of the sample and reagent streams to arriving at the optical cell should be longer than the characteristic time for diffusive mixing.

The hydrodynamic pressure drop (*p_i_*) for a given channel segment (*i*) is given by the Hagen–Poiseuille equation:

*p_i_* = 8*μL_i_Q_i_*/π*r_i_*^4^, (1)

where *μ* is the dynamic viscosity of the fluid, *L_i_* is channel length, *Q_i_* is the flow rate, and *r_i_* is the hydraulic radius [28], which is approximated by *xy*/(*x* + *y*) for a rectangular channel cross-section, where *x* and *y* are the channel width and channel depth, respectively. If we consider the chip design in Figure 1, the sample and reagent flow separately through the serpentine channels (s) and together through the mixing (m), and waste (w) channels; *Q_m_* = *Q_w_* = *Q_s_* + *Q_r_*. The chip was designed to operate at different sample/reagent flow ratios, *R* = *Q_s_*/*Q_r_*, to tune the sensitivity to chlorine. In principle, *p_s_* > *p_m_* + *p_w_* to avoid risk of backflow. For our design, *p_s_* ~ 3–5 kPa, *p_m_* ~ 1.2 kPa, and *p_w_* ~ 0.5 kPa, meeting this condition.

The residence time *t_m_* in the mixing channel is given by *V_m_*/*Q_m_*, where *V_m_* is the volume of the channel. The characteristic time for diffusive mixing (perpendicular to the laminar flow) can be estimated by *t* = *x*^2^/*D*, where *x* and D (~10^−9^ m^2^/s) are the channel width and diffusion coefficient (MO or PR), respectively. To achieve mixing prior to the optical cell, *t_m_* > *t*. Based on the total flow rate used (10 μL/min), the residence time in the mixing channel was approximately 20 s, which was greater than the calculated diffusion time (14 s). This analysis was conservative, as it ignored the actual flow profile of the two laminar streams and the residence time in the first through-hole, which would accelerate mixing. In practice, we observed complete mixing and concluded that the chip design successfully delivered a homogeneous stream to the optical cell.

The chip design and operational protocol, shown in Table 1, used micro-volume samples per measurement. The chip required a 220 μL sample, 15 μL MO (pH 7), 15 μL MO (pH 4), and 30 μL PR for a complete measurement cycle, or less than 35 mL (8.2 mL MO (pH 7); 8.2 mL MO (pH 4); 16.4 mL PR) of each reagent for three months of operation at six cycles per day.

### 3.2. Active Chlorine

Photometric analysis of the bleaching of MO by active chlorine has many practical advantages for our application. MO is widely available, has a low toxicity, and is highly stable. In addition, the proposed method has negligible interference from iron, manganese and nitrite, which are known to interfere with other methods for determining active chlorine. Experiments revealed > 6 months stability under ambient temperature, low temperature (4 °C), direct sunlight, and atmospheric oxygen.

Literature [29] suggests that the analysis of active chlorine using this method should be carried out at an acidic pH. In this study, we modified the method to avoid interference from bound chlorine (chloramines). We used an unbuffered MO solution containing NaBr. Due to the high buffering capacity of swimming pool samples, the pH of the MO-sample mixture remained close to neutral (pH 6–8). In this pH range, active chlorine reacts with bromide to form active bromine, which quantitatively reacts with MO, and bound chlorine does not interfere. The optimization of the bromide concentration revealed that 1000 ppm was most suitable for the application.

**Figure 4:**
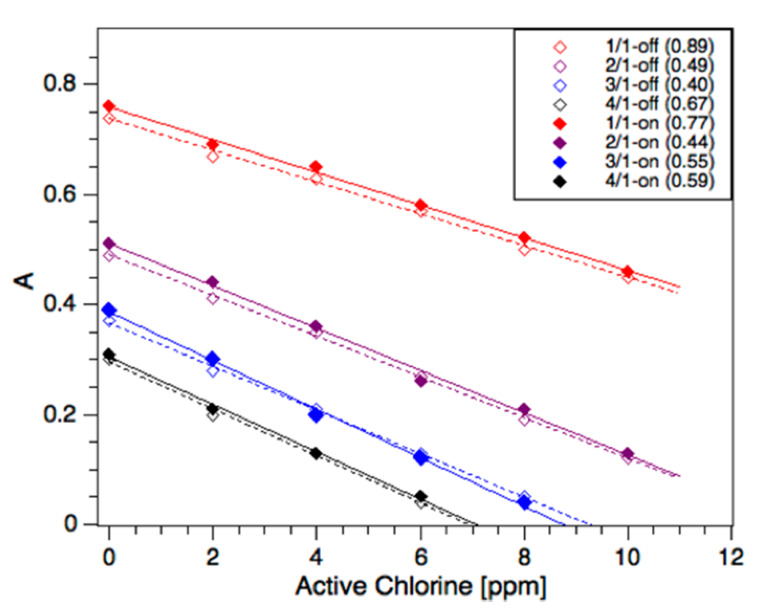
Active chlorine calibrations using sodium hypochlorite standards and 100 ppm methyl orange at neutral pH. Sample/methyl orange flow and volume ratios (S/MO) are indicated in the legend, with notation “off” and “on” referring to off-chip and on-chip experiments, respectively. The legend includes the calculated Limit of Detection (LoD) in parentheses for each respective calibration.

The absorbance of methyl orange strongly depends on the pH of the solution [26] As shown in Figure 2, the acidification of the dye solution in bulk showed bathochromic shifts in the MO spectra, whereas it remained unchanged under alkaline conditions. In our studies, the detection wavelength was set at the isosbestic point of methyl orange at 469 nm to negate minor fluctuations in the pH during the analysis, despite the pH being buffered in swimming pool water. The following results report “on-chip” and “off-chip” (bulk) analyses of active chlorine using the method described above. The reaction between the active chlorine sample (S) and 100 ppm MO was carried out using S/MO volume ratios of 1:1, 2:1, 3:1, and 4:1 (open symbols), as shown in Figure 4. On-chip experiments were carried out using the same ratios, which were achieved by varying the relative flow rates at a fixed total flow of 10 μL/min. This total flow rate allowed for sufficient mixing time (16 s) before entering the optical cell, as shown in the Chip Function section. A good agreement between the on-chip and off-chip methods was obtained, with improved agreement with increasing S/MO ratios and the Limit of Detection (LoD) below 1 ppm active chlorine, as shown in Figure 4. The reason for this is unclear; however, we found that higher S/MO ratios (3:1) were well-suited to chlorine concentrations < 8 ppm (as typically found in swimming pools) and had practical advantages, including the storage of lower volumes of reagent.

The ability to tune the flow ratio enabled the sensitivity and range of measurements to be varied, depending on the sample. The lower ratio (1:1) was, therefore, only used for overdosed pools (>8 ppm), as shown later.

### 3.3. Total Chlorine

**Figure 5:**
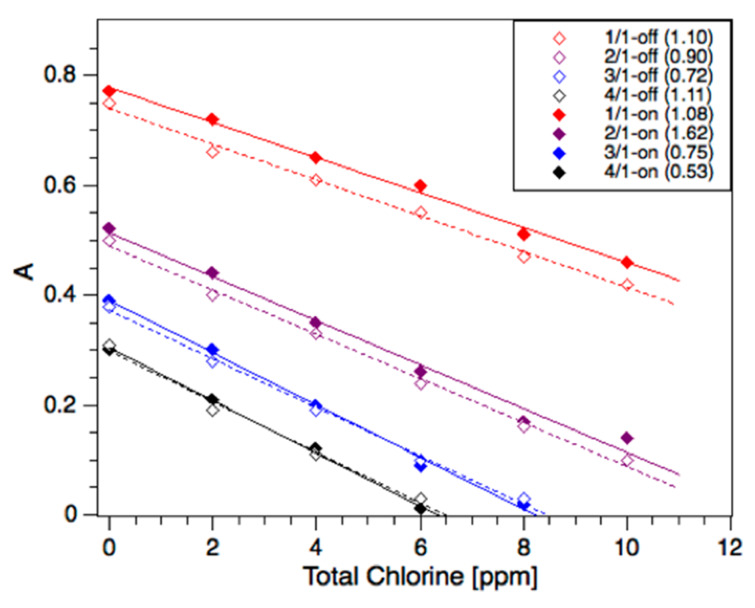
Total chlorine calibrations using sodium hypochlorite standards and 100 ppm methyl orange buffered at pH 4. Sample/methyl orange flow and volume ratios (S/MO) are indicated in the legend, with notation “off” and “on” referring to off-chip and on-chip experiments, respectively. The legend includes the calculated Limit of Detection (LoD) in parentheses for each respective calibration.

Active and bound chlorine-collectively “total” chlorine-react with MO at low pH in the presence of NaBr. In this study, we prepared a MO solution (pH 4–4.5, citrate buffer; 4000 ppm NaBr) that achieved a fast and quantitative reaction. Figure 5 shows the calibrations at different S/MO at pH 4 for on-chip and off-chip experiments. A comparative analysis of the absorbance changes and dilution effects as a function of the added chlorine showed an adjustment to the reaction stoichiometry, similar to previous studies on active chlorine. The highest S/MO ratio (4:1) led to the highest sensitivity, but only over a small concentration range (0–6 ppm). The calculated LoDs are consistent with the increased sensitivity to chlorine at higher mixing ratios during on-chip detection. Therefore, the S/MO ratio of 3:1 was preferred for measurements of real pool samples (<8 ppm).

### 3.4. pH Analysis

The photometric pH analysis exploited the pH-sensitive spectrum of the acid-base indicator, phenol red (PR). PR has two distinct absorbance bands at 430 nm (A432) and 560 nm (A560) in the visible range, which change dramatically between pH 6 (yellow) and pH 8 (pink/red) and are separated by an isosbestic point at 479 nm, as shown in Figure 3 [30]. PR is not currently used in an online pH monitor, possibly due to reagent consumption. In a microfluidic device, the reagent consumption can be small, even when used for many measurements over long periods of time (as discussed here).

**Figure 6:**
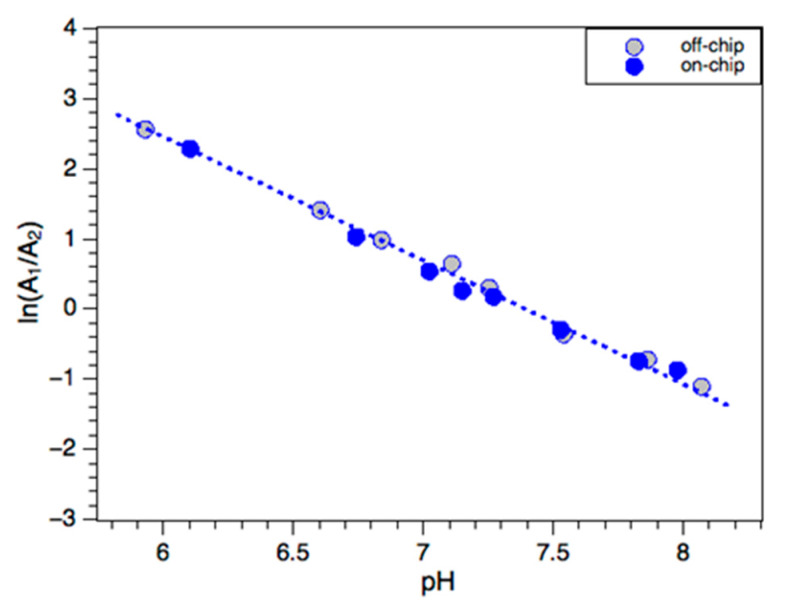
Calibration for on-chip and off-chip pH analysis using phenol red (A1 = 432 nm, A2 = 560 nm).

Figure 6 shows the natural logarithm of the relative peak intensities (A432/A560) in the pH range 6.0–8.5. These calibration samples were prepared using an outdoor domestic swimming pool sample (initial pH 7.3), which was spiked with 0.5 M HCl or 0.5 M NaOH solutions to tune the pH over the calibration range. The S/PR volume (off-chip) or flow (on-chip) ratio was 1:1. A good agreement between the photometric method and a pH electrode was obtained. When the pH was below 6 or above 8.5, the linear relationship was not observed. This is due to either A432 or A560 approaching zero. However, these limits would appear in the pH sensor as values that exceed high or low limits, alerting the user to the need for pool maintenance.

### 3.5. Testing Swimming Pools

To validate the microfluidic swimming pool sensor, we tested samples obtained from 12 swimming pools (nine domestic, two indoor public, and one outdoor public). Samples 11 and 12 were collected and measured on multiple occasions. Every sample had its own ambient situation and sanitation history, thus representing a realistic challenge for the sensor. Examples included a recently purchased property, high organic load (e.g., leaf and animal litter), frequent public use, indoor pools, and various chlorination methods. Owners and operators were not asked to provide any information about their pools prior to analysis. All samples were tested for active chlorine, total chlorine, and pH using photometric (on-chip and off-chip) and other methods discussed below.

**Figure 7:**
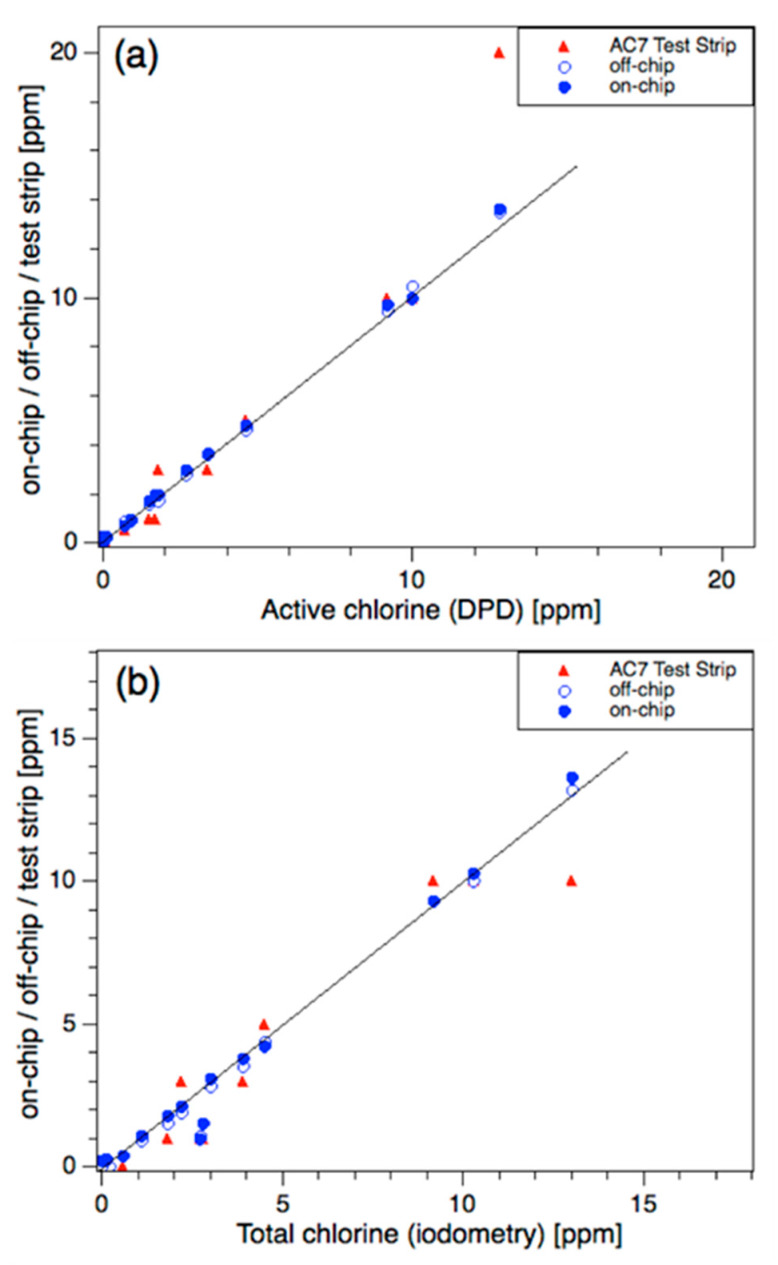
Comparison of (**a**) active chlorine and (**b**) total chlorine measurements against DPD and iodometry results, respectively. The open and closed blue symbols represent the off-chip and on-chip methods, respectively. The red symbols represent the AC7^®^ test strips.

**Figure 8:**
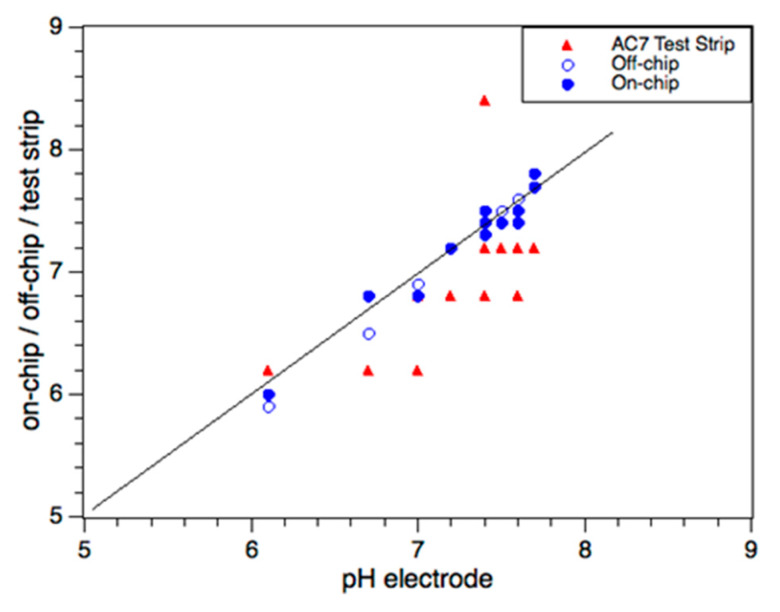
Comparison of pH measurements using on-chip, off-chip, and test strip methods plotted against precision pH electrode results. The open and closed blue symbols represent the off-chip and on-chip methods, respectively. The red symbols represent the AC7^®^ test strips.

Commercially available universal test strips AC7^®^ from AquaCheck (HACH, USA) were used to read the pH and the active and total chlorine levels from the colour table. For greater accuracy, commercial DPD1^®^ (N,N′-diethyl-p-phenylenediamine, HACH, USA) powder pillows were used (bulk analysis) for active chlorine. Total chlorine was analysed by iodometric titration. The measurement of pH was carried out using a precise laboratory-grade pH electrode. For details of these measurement methods, see Experimental. Figure 7 and Figure 8 compare the on-chip and off-chip results with the benchmark results for all 12 swimming pools. In general, there is good agreement between the on-chip and benchmark results. However, for AC7^®^ test strips, the large concentration steps in the colour chart (active chlorine: 0, 0.5, 1, 3, 5, 10, 20 ppm) led to significant scatter in these measurements.

The on-chip analysis of active chlorine was in good agreement with the DPD1^®^ benchmark. For total chlorine, all samples except 3 and 4 agreed with the iodometric titration. Iodometry is non-specific for chlorine, with manganese, iron, and other oxidants known to interfere. Inductively coupled plasma mass spectroscopy (ICP-MS) reported high average concentrations (>100 ppm) of Na and Ca, as expected, and low concentrations of K (5–16 ppm), B (0–7 ppm), Mg (4–34 ppm) and Si (<5 ppm). No manganese, iron, or copper was found, suggesting that any interfering oxidants (assuming they were present) were probably organic, perhaps originating from the local environment. This could not be proven experimentally but is supported by the good correlation between the on-chip analysis and the other methods used.

As shown in Table 2, only six samples had chlorine levels in range (1–4, 6, 12). Three of these were professionally managed (1–3). Therefore, most of the domestic pools were either over- or under-dosed with chlorine. This highlights the need for an effective online chlorine sensor for domestic pools. Three pools were over-dosed in the range of 4 to 14 ppm (5, 7 and 11). For more than 8 ppm, the S/MO ratio was switched from 3:1 to 1:1 to widen the measurement range. Samples 8, 9, 10, 11c, shown in Table 2, were under-dosed and, in Sample 10, bound chlorine was the major chlorine species (0.4 ppm on-chip). In one instance (sample 11), the owners had recently purchased the property and were unaware that their chlorinator had failed. The pool was manually dosed prior to purchase (11a) and degraded over two weeks (11b,11c) to near zero chlorine.

Finally, we considered the pH measurements. The on-chip method showed good agreement (<0.2 difference) with the precision laboratory pH electrode, as shown in Figure 8 and Table 2. All analysis methods reported that some pools were slightly acidic, compared to the ideal swimming pool pH range (7.2–7.8). The AC7 test strips showed the highest differences compared to the pH electrode, with significant scatter, as shown by the red symbols in Figure 8. This inaccuracy stems from the visual comparison with the supplied colour chart, which is in steps of 0.4 or 0.6 pH units.

## 4. Conclusions

We report on a microfluidic swimming pool sensor based on photometric analysis of MO and PR dye. The chip contained a 2.2 mm path length optical cell that was capable of detecting active chlorine, total chlorine, and pH using microliter sample and reagent volumes per measurement. Based on the current design, the sensor uses less than 35 mL of reagent solution over a summer season (three months, six measurements per day). The MO isosbestic point is used to avoid pH effects on the chlorine measurements. Changing the sample/reagent flow ratio permits the tuning of the concentration range and sensitivity of the analysis, which is shown to be useful for over-chlorinated pools (e.g., after breakpoint chlorination). For PR, the ratio of absorbance, measured at 430 nm and 560 nm, was used over the pH range of 6 to 8.5. The on-chip results showed excellent agreement with a precision laboratory pH electrode and outperformed single-use test strips, which are often used for domestic pool monitoring.

Twelve swimming pools were sampled (nine domestic, two indoor public, and one outdoor public) and analysed using the on-chip chlorine and pH methods. The results showed that the on-chip method matched the performance of high-precision analytical methods and out-performed low-cost alternatives. No pre-treatment of the pool samples was necessary in this study. It was found that approximately half of the samples were over- or under-chlorinated, posing a health risk to the (domestic) users. In most cases, the owners of the pools were unaware of the imbalanced chemical condition of the pool. In one case, the owner was unaware that their chlorinator was not working at all. These examples highlight the need for a low-cost and automated online microfluidic sensor.

## Figures and Tables

**Table 1:** On-chip measurement protocol.

Protocol Step	Time (min)	Flow Rate (μL/min)			
		S	MO pH 7	MO pH 4	PR
Sample Flush	2	50	0	0	0
Active Cl (<8 ppm) ^#^	6	7.5	2.5	0	0
Total Cl (<8 ppm) ^#^	6	7.5	0	2.5	0
pH	6	5	0	0	5

^#^ For > 8 ppm, the sample to MO ratio was adjusted to 1:1.

**Table 2:** Swimming pool measurements.

ID	Sample Type ^[1]^	Active Chlorine (ppm)				Total Chlorine (ppm)				pH				Notes
		On-chip	Off-chip	DPD1	AC7^®^	On-chip	Off-chip	D2022	AC7^®^	On-Chip	Off-chip	pH electrode	AC7^®^	
1	P/I	0.7	0.9	0.7	0.5	1.1	0.9	1.1	1	6.8	6.9	7	6.8	-
2	P/I	2	1.7	1.8	3	2.1	1.9	2.2	3	7.4	7.5	7.6	6.8	-
3	P/O	1	0.9	0.9	1	1	1.1	2.7	1	6.8	6.9	7	6.2	-
4	D/O	1.7	1.6	1.5	1	1.5	1.5	2.8	1	7.5	7.4	7.4	8.4	-
5	D/O	10	10.5	10	10	10.3	10	10.3	10	7.3	7.5	7.4	6.8	High Cl
6	D/O	2	1.8	1.7	1	1.8	1.5	1.8	1	7.4	7.4	7.4	7.2	-
7	D/O	4.8	4.6	4.6	5	4.2	4.4	4.5	5	7.4	7.5	7.5	7.2	High Cl
8	D/O	−0.2	0	−0.1	0	−0.3	0	0.2	0	7.5	7.6	7.6	7.2	Low Cl
9	D/O	0.3	0	−0.1	0	0.2	0	0	0	7.5	7.5	7.6	7.2	Low Cl
10	D/O	0.1	0	0	0	0.4	0.4	0.6	0	7.8	7.8	7.7	7.2	Low Cl
11a	D/O	13.6	13.5	12.8	20	13.6	13.2	13	10	7.2	7.2	7.2	6.8	High Cl
11b	D/O	9.7	9.5	9.2	10	9.3	9.3	9.2	10	7.4	7.5	7.5	7.2	High Cl
11c	D/O	0.2	0.3	0.1	0	0.3	0.2	0.1	0	7.7	7.7	7.7	7.2	Low Cl
12a	D/O	3.6	3.7	3.4	3	3.8	3.5	3.9	3	6.8	6.5	6.7	6.2	-
12b	D/O	3	2.8	2.7	3	3.1	2.8	3	3	6	5.9	6.1	6.2	-

^[1]^ D = domestic; P = public; I = indoor; O = outdoor
